# Supplementary material for: Novel Driver Strength Index highlights important cancer genes in TCGA PanCanAtlas patients
Source: PeerJ. 2022 Aug 11;10:e13860. doi: 10.7717/peerj.13860 (PMC9375969; doi:10.7717/peerj.13860)
Supplement: Supplemental Information 2 [file peerj-10-13860-s002.zip › COHORTS/genes plots/detailed/2021_8_16_14_9_distribution_events_detailed_20.pdf]

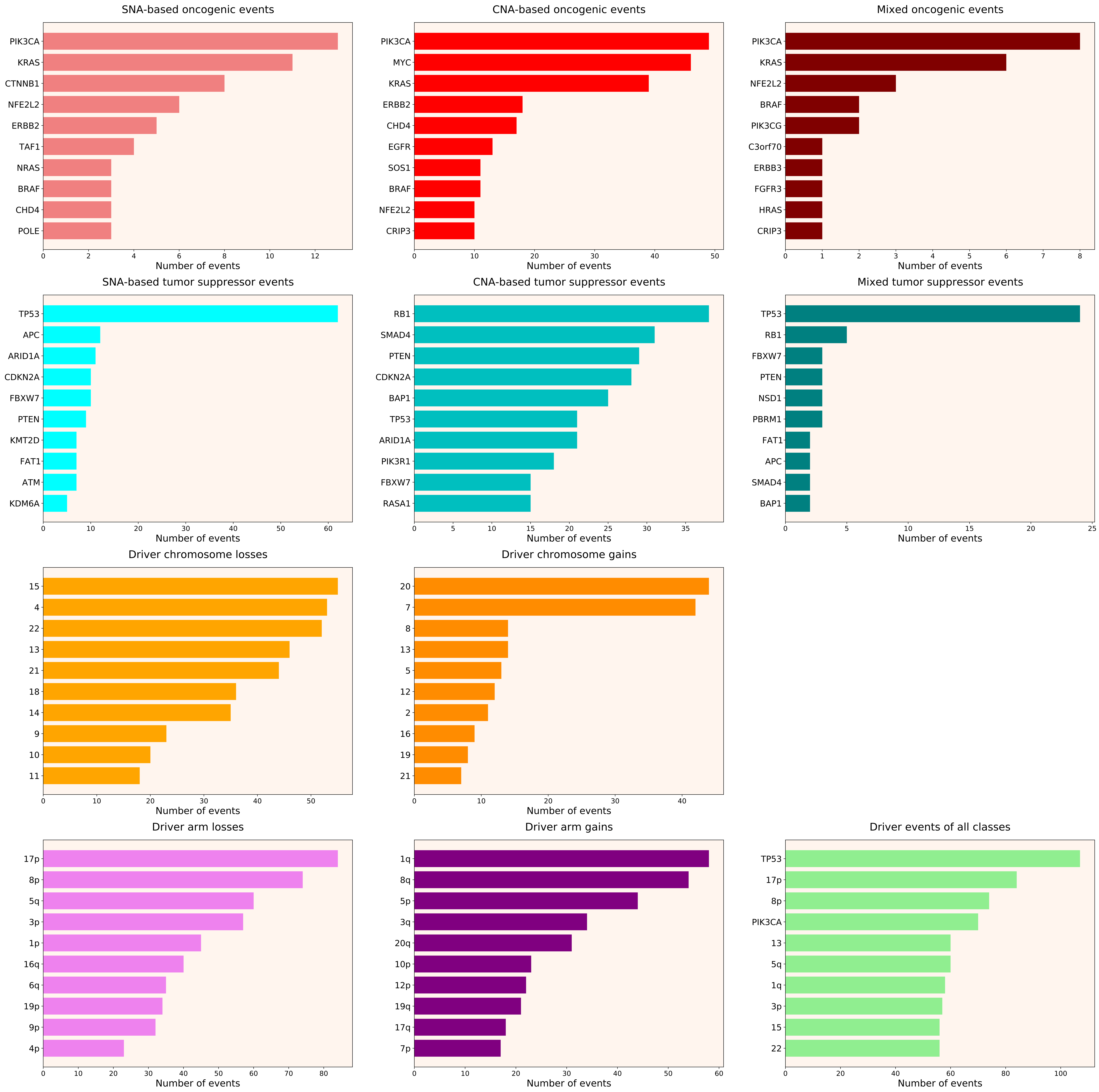
SNA-based oncogenic events
PIK3CA
KRAS
CTNNB1
NFE2L2
ERBB2
TAF1
NRAS
BRAF
CHD4
POLE
0 2 4 6 8 10 12
Number of events
CNA-based oncogenic events
PIK3CA
MYC
KRAS
ERBB2
CHD4
EGFR
SOS1
BRAF
NFE2L2
CRIP3
0 10 20 30 40 50
Number of events
Mixed oncogenic events
PIK3CA
KRAS
NFE2L2
BRAF
PIK3CG
C3orf70
ERBB3
FGFR3
HRAS
CRIP3
0 1 2 3 4 5 6 7 8
Number of events
SNA-based tumor suppressor events
TP53
APC
ARID1A
CDKN2A
FBXW7
PTEN
KMT2D
FAT1
ATM
KDM6A
0 10 20 30 40 50 60
Number of events
CNA-based tumor suppressor events
RB1
SMAD4
PTEN
CDKN2A
BAP1
TP53
ARID1A
PIK3R1
FBXW7
RASA1
0 5 10 15 20 25 30 35
Number of events
Mixed tumor suppressor events
TP53
RB1
FBXW7
PTEN
NSD1
PBRM1
FAT1
APC
SMAD4
BAP1
0 5 10 15 20 25
Number of events
Driver chromosome losses
15
4
22
13
21
18
14
9
10
11
0 10 20 30 40 50
Number of events
Driver chromosome gains
20
7
8
13
5
12
2
16
19
21
0 10 20 30 40
Number of events
Driver arm losses
17p
8p
5q
3p
1p
16q
6q
19p
9p
4p
0 10 20 30 40 50 60 70 80
Number of events
Driver arm gains
1q
8q
5p
3q
20q
10p
12p
19q
17q
7p
0 10 20 30 40 50 60
Number of events
Driver events of all classes
TP53
17p
8p
PIK3CA
13
5q
1q
3p
15
22
0 20 40 60 80 100
Number of events
